# The epithelial-mesenchymal transition phenotype of metastatic lymph nodes impacts the prognosis of esophageal squamous cell carcinoma patients

**DOI:** 10.18632/oncotarget.9036

**Published:** 2016-04-27

**Authors:** Jing Wen, Kong-Jia Luo, Qian-Wen Liu, Geng Wang, Mei-Fang Zhang, Xiu-Ying Xie, Hong Yang, Jian-Hua Fu, Yi Hu

**Affiliations:** ^1^ State Key Laboratory of Oncology in South China, Collaborative Innovation Center for Cancer Medicine, Sun Yat-sen University Cancer Center, Guangzhou 510060, China; ^2^ Guangdong Esophageal Cancer Institute, Guangzhou 510060, China; ^3^ Department of Thoracic Oncology, Sun Yat-sen University Cancer Center, Guangzhou 510060, China; ^4^ Department of Thoracic Surgery, Cancer Hospital of Shantou University Medical College, Shantou 515041, China; ^5^ Department of Pathology, Sun Yat-sen University Cancer Center, Guangzhou 510060, China

**Keywords:** esophageal squamous cell carcinoma, epithelial-mesenchymal transition, metastatic lymph node, prognosis

## Abstract

Epithelial-mesenchymal transition (EMT) plays a key role in tumor metastasis, but the significance of EMT phenotype to the prognosis of esophageal squamous cell carcinoma (ESCC) patients remains unclear. We used immunohistochemistry to examine the expression of the EMT-related proteins E-cadherin, N-cadherin and vimentin in samples of T3N1-3M0 ESCC from 155 primary tumors (PTs) with paired metastatic lymph nodes (MLNs) and 58 PTs without paired MLNs. Based on the expression pattern of the EMT markers, PTs and MLNs were classified as EMT wild, hybrid, null or complete type. The hybrid (42.7%) and complete (39.4%) types predominated among PTs, whereas the wild (34.2%) and hybrid (52.9%) types predominated among MLNs, and EMT phenotypes differed between the paired PTs and MLNs (*P* < 0.001). Univariate analysis revealed that, for PTs, the EMT phenotype was associated with N-stage (*P* = 0.039) but not patient survival, and that patients with complete or hybrid type MLNs had better overall survival (OS, *P* = 0.001) and disease-free survival (DFS, *P* = 0.005) than patients with null and wild type MLNs, especially those with N1-stage disease (*P* = 0.017 for OS, and *P* = 0.017 for DFS, respectively). Multivariate analysis revealed that wild and null type MLNs as well as older age and N2-3 stage were independent predictors of OS and DFS (*P* < 0.05). Thus MLNs exhibit EMT phenotypes that are distinct from those of their PT and may serve as a novel independent prognostic indicator in ESCC.

## INTRODUCTION

In China, esophageal squamous cell carcinomas (ESCCs) account for most malignant esophageal tumors [1]. Moreover, most patients are at an advanced stage with lymph node metastasis when first diagnosed [2]. Due to the heterogeneity of tumor cells at the primary site or selection pressure during tumor metastasis, genotypes and gene and protein expression may differ between the primary tumor (PTs) and metastatic lymph nodes (MLNs) in ESCCs, as well as other cancer types [3–7]. These underlying molecular differences lead to differences in the biological characteristics of PTs and their metastases. This raises the question, should the molecular characteristics of the PT be used to determine therapy or predict prognosis [8]?

Cancer metastasis occurs through a series of steps [9]. Epithelial-mesenchymal transition (EMT) is thought to be critical for the initial transformation from a benign to an invasive phenotype, while its reverse process, mesenchymal-epithelial transition (MET), is an important contributor to the later stages of metastasis formation [10]. A hallmark of EMT is the down-regulation of E-Cadherin accompanied by increased expression of mesenchymal neural cadherin (N-cadherin). This “cadherin switch” alters cell adhesion. In addition, activation of vimentin expression, which alters the composition of intermediate filaments, is frequently used as a mesenchymal marker in cancer [11].

Although EMT is often portrayed simply as a gain of mesenchymal markers coupled with a loss of epithelial features, in reality it usually produces cells residing within a spectrum of intermediate phenotypic states – often termed a “partial EMT” – where they co-express newly acquired mesenchymal markers together with retained epithelial ones [12]. Given the different roles of EMT and MET in PTs and metastatic sites and the existence of partial EMT status in ESCC, it is imperative to understand the EMT phenotypes in ESCC PTs and MLNs. In this study, therefore, we compared the EMT phenotypes of PTs and their paired MLNs based on the expression pattern of EMT-related proteins and investigated the clinical significance of each phenotype in a series of resected T3N1-3M0 ESCCs.

## RESULTS

### Expression of EMT markers in ESCC PTs and MLNs

Complete staining information for three EMT markers, E-cadherin, N-cadherin and vimentin, was obtained in a total of 155 PTs paired with MLNs and 58 PTs without paired MLNs. Among these, the E-cadherin expression in 134 paired PTs and MLNs was reported previously [13]. The tissue microarray cores from 8 pairs of PTs and MLNs and 13 PTs without paired MLNs were excluded from our analysis because they were dropped during the immunohistochemistry (IHC) procedures and so could not be used.

**Figure 1:**
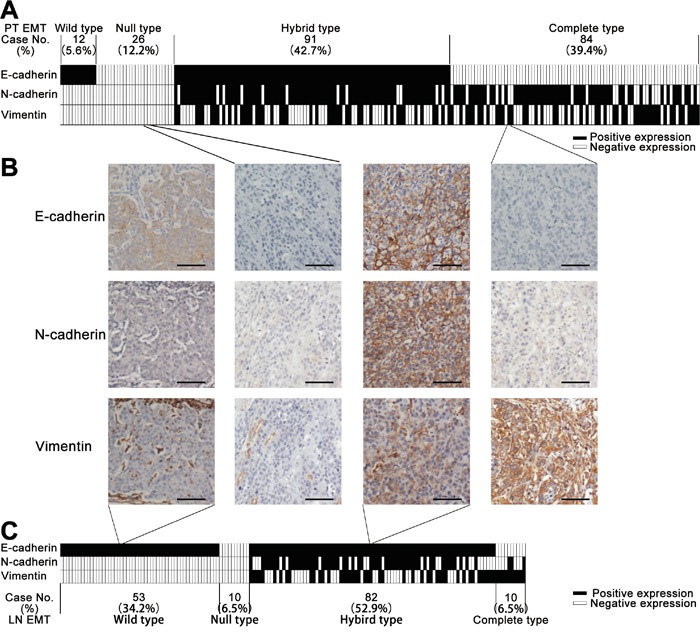
**A.** Classification of the epithelial-mesenchymal transition (EMT) phenotype of 213 primary esophageal squamous cell carcinoma (ESCC) tumors (PTs) based on the pattern of their E-cadherin, N-cadherin and vimentin expression. Each column represents one case. Columns in black represent positive expression, white negative expression. **B.** Representative cases of each EMT phenotype with their corresponding immunostaining of E-cadherin, N-cadherin and vimentin. Scale bar, 100 μm. **C.** Classification of the EMT phenotype for 155 ESCC metastatic lymph nodes (MLNs) based on the patterns of their E-cadherin, N-cadherin and vimentin expression. Each column represents one case. Columns in black represent positive expression, white negative expression.

The positive staining of E- and N-cadherin was localized to the cellular membrane, while vimentin was in the cytoplasm (Figure 1B). Positive expression of E-cadherin, N-cadherin and vimentin was detected in 48.4% (103/213), 65.3% (139/213) and 43.7% (93/213) of PTs, respectively, but in 87.1% (135/155), 38.1% (59/155) and 35.1% (55/155) MLNs. Significantly increased E-cadherin (*P* < 0.001) positive staining and decreased N-cadherin (*P* < 0.001) and vimentin (*P* = 0.036) positive staining was observed in the 155 MLNs as compared to their paired PTs (McNemar Chi-square test; Supplementary Table S1). No significant association was found between expression of these EMT markers and the patients' clinicopathological variables (*P* > 0.05, Chi-square or Fishers' exact test; Supplementary Table S2).

### EMT phenotypes in ESCC PTs and MLNs

Based on the combined IHC results for the epithelial (E-cadherin) and mesenchymal (N-cadherin and vimentin) markers from our tissue microarrays, 12 (5.6%) wild, 26 (12.2%) null, 91 (42.7%) hybrid and 84 (39.4%) complete types were identified among the 213 PTs, while 53 (34.2%) wild, 10 (6.5%) null, 82 (52.9%) hybrid and 11 (6.5%) complete types were identified among the 155 MLNs (Figure 1A, 1C). In addition, the EMT phenotypes differed significantly between the 155 PTs and their paired MLNs (*P* < 0.001, McNemar-Bowker test; Table 1). Furthermore, the EMT phenotypes of the PTs were significantly associated with N-stage (*P* = 0.039 by Chi-square test; Supplementary Table S3), whereas there was no significant association between the EMT phenotypes of PTs or MLNs and other clinicopathological parameters (*P* > 0.05, Chi-square or Fishers' exact test; Supplementary Table S3).

**Table 1:** Classification of EMT phenotypes in paired primary tumors and metastatic lymph nodes of 155 Stage T3N1-3M0 ESCCs

		Paired metastatic lymph nodes				Total (%)	*P^a^*
		Wild type (%)	Null type (%)	Hybrid type (%)	Complete type (%)		
Primary tumors	Wild type	2 (25.0)	1 (12.5)	4 (50.0)	1 (12.5)	8 (5.2)	**< 0.001**
	Null type	9 (47.4)	1 (5.3)	8 (42.1)	1 (5.3)	19 (12.3)	
	Hybrid type	22 (35.5)	4 (6.5)	31 (50.0)	5 (8.1)	62 (40.0)	
	Complete type	20 (30.3)	4 (6.1)	39 (59.1)	3 (4.5)	66 (42.6)	
Total (%)		53 (34.2)	10 (6.5)	82 (52.9)	10 (6.5)	155 (100)	

a McNemar-Bowker test.

### Impact of EMT phenotype on patient survival

Among the 213 ESCC patients, the median survival time was 18.7 months (range, 1.6-178.3 months), with 184 cancer-related deaths. Kaplan-Meier analysis revealed that increased expression of vimentin in MLNs but not PTs significantly improved overall survival (OS, *P* = 0.014; Supplementary Figure S1I) but not disease-free survival (DFS, *P* = 0.124; Supplementary Figure S1L). However, expression of neither E-cadherin nor N-cadherin in PTs or MLNs significantly correlated with OS or DFS (*P* > 0.05; Supplementary Figure S1).

**Figure 2:**
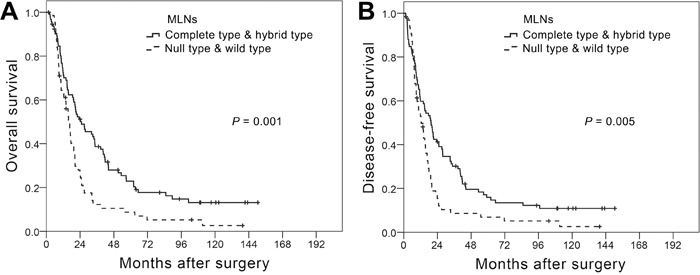
Kaplan-Meier curves for overall survival **A.** and disease-free survival **B.** among T3N1-3M0 ESCC patients differentiated based on metastatic lymph node (MLN) epithelial-mesenchymal transition (EMT) phenotype. Curves were compared using the log-rank test.

**Figure 3:**
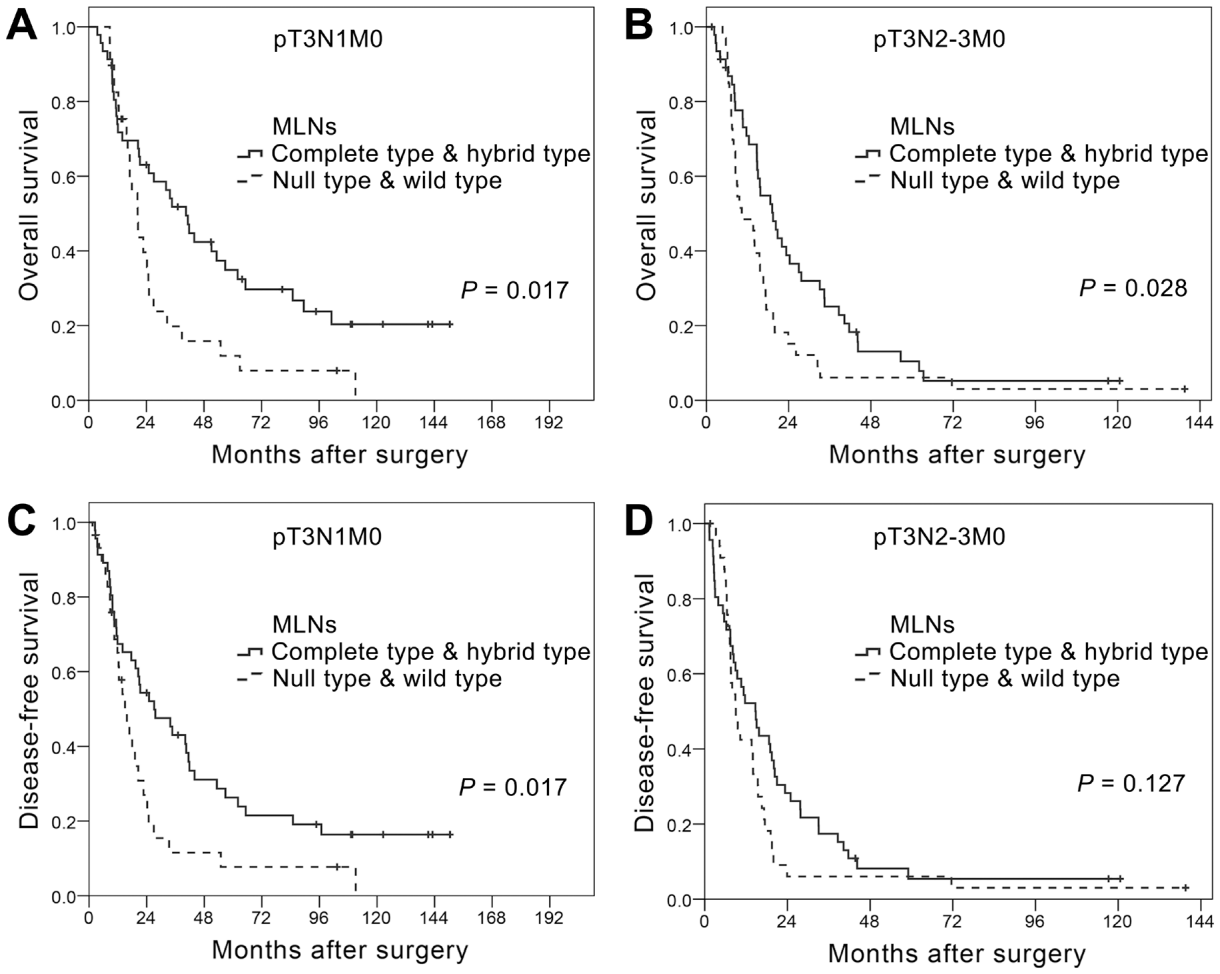
Kaplan-Meier curves for overall survival **A.** and **B.** and disease-free survival **C.** and **D.** among T3N1M0 (A and C) and T3N2-3M0 (B and D) ESCC patients differentiated based on metastatic lymph nodes (MLN) epithelial-mesenchymal transition (EMT) phenotype. Curves were compared using the log-rank test.

When we then assessed survival, taking EMT phenotypes into consideration, we detected no significant difference in OS or DFS among the EMT phenotypes of PTs (*P* = 0.944 for OS and *P* = 0.607 for DFS; Supplementary Figure S2A, S2B and Supplementary Table S4). However, different MLN EMT phenotypes were associated with significantly different OS and DFS (*P* = 0.001 for OS and *P* = 0.008 for DFS; Supplementary Figure S2C, S2D and Supplementary Table S4). The survival curves for patients with wild or null type MLNs (negative for mesenchymal markers) were similar to each other, as were the curves for patients with complete or hybrid type MLNs (positive for mesenchymal markers). When we therefore combined the wild and null types and the complete and hybrid types for survival analysis, we found that the complete/hybrid group showed better OS and DFS than the null/wild group (*P* = 0.001 for OS and *P* = 0.005 for DFS. Figure 2A, 2B). Moreover, the differences in OS (Figure 3A, 3B) and DFS (Figure 3C, 3D) between the two combined groups were noticeably greater in N1 than N2-3 patients.

In addition to EMT phenotype, patients' age (*P* = 0.004) and N-stage (*P* = 0.009) correlated significantly with OS in a univariate survival analysis (Supplementary Table S4). Similarly, significantly poorer DFS was observed in patients older than 58 years (*P* = 0.010) or at N2-3 stage (*P* = 0.010, Supplementary Table S4). Tumor differentiation correlated significantly with DFS (*P* = 0.046) but not OS (*P* = 0.196, Supplementary Table S4). No significant correlations with survival were found for any other clinicopathological variables, including patients' gender, tumor location, and length (*P* > 0.05; Supplementary Table S4). Multivariate analysis using the Cox proportional hazards regression model with factors significantly affecting survival determined by univariate analysis showed that wild/null type, age ≥ 58 years and N2-3 stage were all independent predictors of poorer OS and DFS in T3N1-3M0 ESCCs (*P* < 0.05; Table 2).

**Table 2:** Multivariate Cox proportional hazards regression model predicting survival in 155 T3N1-3M0 esophageal squamous cell carcinomas

Variables	Category	Overall survival			Disease-free survival		
		HR	95% CI	*P*	HR	95% CI	*P*
Age (years)	< 58*^a^*	1	Reference		1	Reference	
	≥ 58	1.711	1.190-2.461	**0.004**	1.498	1.055-2.127	**0.024**
N-stage	N1	1	Reference		1	Reference	
	N2-3	2.017	1.423-2.860	**<0.001**	1.809	1.287-2.542	**0.001**
MLN' EMT phenotypes	Complete and hybrid type	1	Reference		1	Reference	
	Wild and null type	1.737	1.222-2.469	**0.002**	1.580	1.117-2.234	**0.010**

a Median age.

MLN, metastatic lymph nodes; EMT, epithelial-mesenchymal transition; HR, hazard ratio; CI, confidence interval.

## DISCUSSION

In this study, we classified the EMT phenotypes of PTs and MLNs in a cohort of T3N1-3M0 ESCC patients based on the expression pattern of EMT-related markers. The classification of EMT phenotypes in ESCC is still controversial, mainly due to the existence of incomplete types. In earlier studies, Usami et al. [14] classified cancers that had both epithelial and mesenchymal characteristics, which corresponds to the hybrid type in the present study, into their complete EMT category. On the other hand, Sung et al. [15] grouped the hybrid and null types, which were positive or negative for both epithelial and mesenchymal markers, into the incomplete type. In the present study, we defined the EMT phenotypes as wild, null, hybrid and complete types, without including in any group phenotypes that exhibited molecular characteristics different from the rest of the group.

We observed a loss of E-cadherin expression in 51.6% of T3N1-3 ESCC PTs and gain of N-cadherin and vimentin expression in 65.3% and 43.7%, respectively. This is consistent with the established roles of EMT during ESCC progression, since reduced E-cadherin [16–20] with increased N-cadherin [21, 22] and vimentin [23, 24] expression would reduce cell adhesion, increase cell mobility, and ultimately enhance cell invasiveness and metastasis to form advanced stage tumors. However, we found no significant association between expression of any EMT marker in PTs and N-stage, a clinical lymph node metastasis parameter. After classifying ESCC PTs into the aforementioned four groups according to their EMT phenotypes, we found that only complete type PTs were associated with a higher probability of advanced N-stage in T3N1-3M0 ESCC patients.

When looking into the EMT markers' expression in MLNs, significantly increased E-cadherin and decreased N-cadherin and vimentin expression were observed in comparison with paired PTs. Accordingly, in MLNs, wild and hybrid phenotypes accounted for a higher proportion of cases than in PTs, where complete and hybrid type predominated. This change affirms the speculation that mesenchymal ESCC cells recruited to target organs undergo a phenotypic reversal from mesenchymal back to epithelial via the MET process [25]. This enables those cells to construct connections to resident normal cells and so survive and form metastases [10].

Both univariate and multivariate analyses revealed associations between patient survival and EMT phenotypes in MLNs but not PTs. The complete and hybrid type MLNs showed similarity and were associated with a better prognosis than the null and wild types. This is contrary to the correlation between the EMT phenotypes of PTs and survival among ESCC patients reported by Sung et al. [15], who found that wild and null type PTs were associated with a better prognosis than the complete and hybrid types. This discrepancy may reflect the different roles of EMT and MET in PTs and MLNs. Complete or hybrid type PTs would be expected to exhibit more aggressive behavior, resulting in a poor prognosis, whereas complete or hybrid type MLNs may be those not going through the MET process. These cells would not be expected to survive or grow as well as those undergoing MET. In addition, it appears that the MLN EMT phenotype does not reflect the extent of disease dissemination at surgery, as it was not significantly associated with N-stage at surgery. We therefore suggest the MET process tumor cells undergo within MLNs may directly reduce patient survival by enhancing further systemic dissemination. However, this speculation requires further verification.

A limitation of this study is that we did not evaluate the EMT phenotype of all MLNs from each patient. Up to now, the lymphatic metastasis route of esophageal tumors has not been clear [26], and the site of MLNs does not appear to be a significant prognostic factor for ESCCs [27]. This made it impossible to select a representative MLN. Therefore, for those with more than one MLN available for use in tissue microarrays, one MLN was chosen at random from each case. On the other hand, we selected the most important and representative markers for EMT-phenotype determination. EMT is a dynamic process, during which changes in the various EMT-related molecules do not occur at the same time [28]. The dynamic and sequential changes in the levels of EMT markers within tumor tissues have not yet been fully elucidated. We anticipate that future determination of all the EMT markers in PTs and MLNs will clarify to some degree the routes of lymph node metastasis.

In conclusion, our findings indicate substantial differences between EMT phenotypes in PTs and their corresponding MLNs in ESCCs. We reported here for the first time that the EMT status of MLNs may serve as an independent predictor of prognosis in ESCC patients after complete surgical resection of T3N1-3M0 tumors, though that finding requires confirmation in larger cohort studies. In addition, identification of specific EMT phenotypes in MLN samples might provide a tool to better stratify and predict patient outcomes.

## MATERIALS AND METHODS

### Study population

The study was approved by the Ethics Committee of Sun Yat-Sen University Cancer Center and performed in accordance with the principles embodied in the Declaration of Helsinki. The study population was retrospectively selected from ESCC patients who had undergone esophagectomy between July 1997 and December 2004 at the Department of Thoracic Surgery. Esophagectomy with standard or extended dissection of thoracic and abdominal lymph nodes was executed in patients with no evidence of distant metastatic disease. Selection criteria were 1) histologic proof of thoracic T3N1-3M0 ESCC according to the 7th edition AJCC TNM staging system, 2) availability of formalin-fixed and paraffin-embedded PT and MLN samples, 3) complete surgical resection, 4) no neoadjuvant or adjuvant treatment, 5) and complete follow-up data. Patients with a history of other cancer or death during the perioperative period were excluded. The patients were followed every month for the first 3 months, every 3 months for the first year, every 6 months for the next 2 years, and then annually. Esophageal endoscopy, thoracic and abdominal computed tomography, bone scans, and cervical ultrasonography (with biopsy if indicated) were adopted when necessary to detect recurrence and/or metastasis.

### Tissue microarray construction

We obtained 234 PT and 3,099 regional LN samples from the 234 selected T3N1-3M0 patients. A median of 12 LNs were resected from each patient. For each sample, hematoxylin and eosin-stained sections from a single randomly selected paraffin block were reviewed to define representative tumor regions – i.e., histologic proof of ESCC and the existence of primary or metastatic lesions with a diameter larger than 3 mm. We then randomly selected from 42 patients one lymph node per case that satisfied the aforementioned criteria. In total, 163 pairs of surgically resected ESCC primary tissues and corresponding MLNs were selected. In addition, 71 PTs without eligible MLNs were also recruited. Tissue microarrays were constructed using a Beecher Instruments tissue microarrayer (Beecher Instruments, Sun Prairie, WI). Three targeted core samples with a 1-mm diameter were punched from each specimen and arrayed on a recipient paraffin block.

### Immunohistochemistry

Sections (4-μm) of the tissue microarray block were cut and placed on glass slides for IHC staining. A mouse monoclonal anti-E-cadherin antibody (Maxim, 1:100 dilution) was used for E-cadherin staining with an Elivision plus IHC kit (Maxim, Fuzhou, China), as described previously [13]. N-cadherin and vimentin were stained using a SPlink Detection kit (ZSGB-BIO, Beijing, China) with rabbit monoclonal anti-N-cadherin (Epitomics, Burlingame, CA, 1:500 dilution) and anti-vimentin (Epitomics, 1:250 dilution) antibodies. A negative control was obtained by replacing the primary antibody with normal rabbit IgG. The expression was recorded as negative if no staining was observed in any array core from the same tissue sample; otherwise, expression was recorded as positive.

### Classification of EMT phenotypes

Based on the combined results from IHC staining of epithelial (E-cadherin) and mesenchymal (N-cadherin and vimentin) markers, each ESCC PT or MLN specimen was classified into one of the following four categories, as described previously [13, 14] wild type (positive for E-cadherin and negative for both mesenchymal markers), null type (negative for both E-cadherin and mesenchymal markers), hybrid type (positive for both E-cadherin and any mesenchymal marker), and complete type (negative for E-cadherin and positive for any mesenchymal marker) [14, 15].

### Statistical analysis

Statistical analysis was performed using SPSS 19.0 (SPSS, Chicago, IL). A McNemar Chi-square or McNemar-Bowker test was used to compare the positive staining proportions of E-cadherin, N-cadherin and vimentin, and the EMT phenotypes between PTs and MLNs. The correlation between EMT markers or phenotypes and clinicopathological parameters was analyzed using the Chi-square or Fishers' exact test. OS was defined as the time from surgery to death from any cause, censoring patients who were still alive at the time of last follow-up. DFS was the time from surgery to regional relapse, distant metastasis or cancer-related death, censoring patients who were still alive without recurrent carcinoma at the last follow-up. Survival curves were analyzed using the Kaplan-Meier method and log-rank test. To determine independent factors significantly related to prognosis, multivariate analysis was performed using the Cox proportional hazards regression model with a forward stepwise procedure and conditional likelihood ratio test. A significant difference was declared if the *P*-value from a two-tailed test was less than 0.05.

## SUPPLEMENTARY FIGURES AND TABLES






