# Supplementary material for: The epithelial-mesenchymal transition phenotype of metastatic lymph nodes impacts the prognosis of esophageal squamous cell carcinoma patients
Source: Oncotarget. 2016 Apr 27;7(25):37581–8. doi: 10.18632/oncotarget.9036 (PMC5122333; doi:10.18632/oncotarget.9036)
Supplement: Supplementary file 1 [file oncotarget-07-37581-s001.pdf]

The epithelial-mesenchymal transition phenotype of metastatic lymph nodes impacts the prognosis of esophageal squamous cell carcinoma patients

SUPPLEMENTARY FIGURES AND TABLES

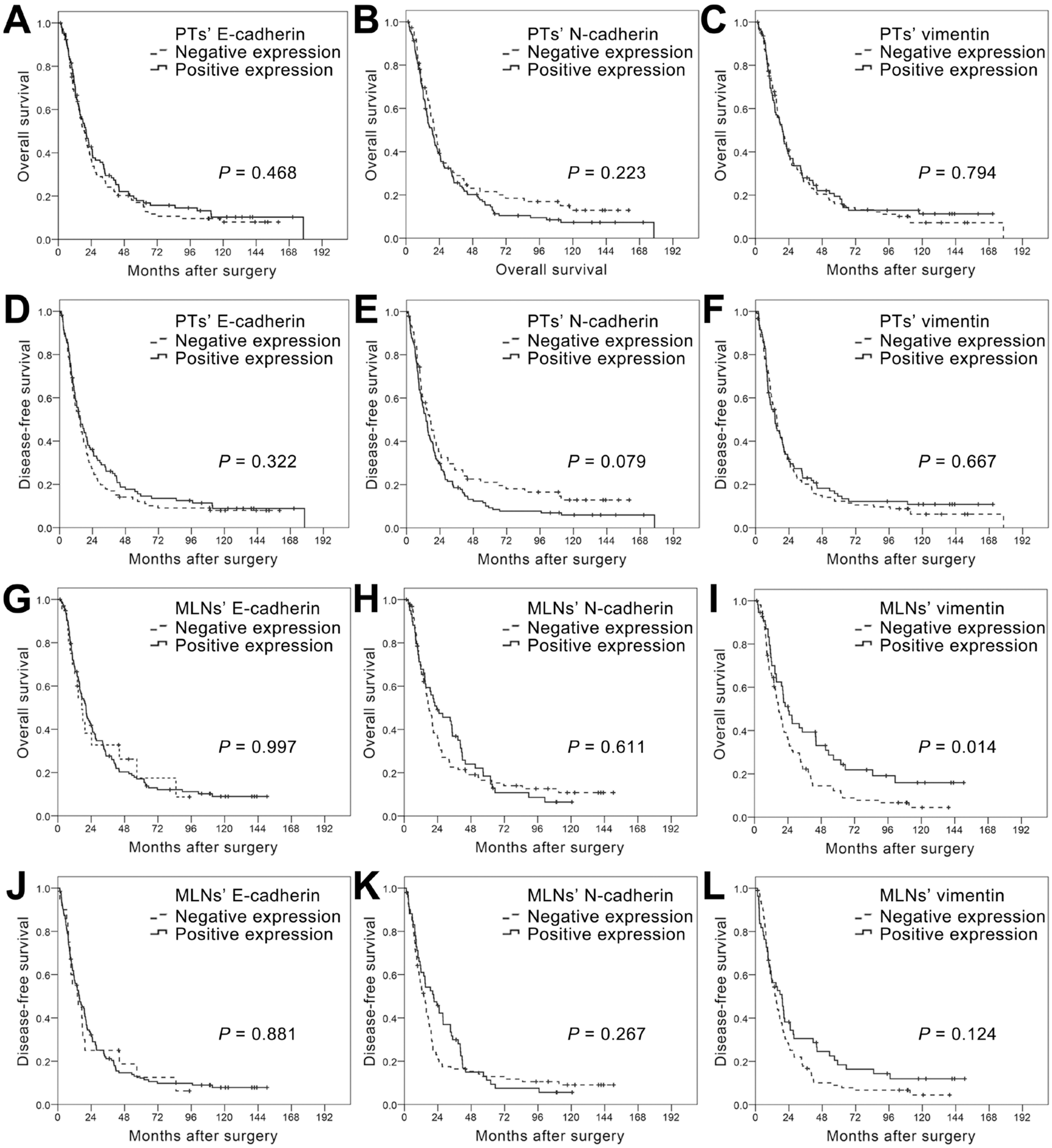

Supplementary Figure S1: Overall survival **A, B, C, G, H, and I.** and disease-free survival **D, E, F, J, K,** and **L.** curves by Kaplan–Meier analysis with log-rank test for T3N1-3M0 esophageal squamous cell carcinoma patients according to primary tumors' (A, B, C, D, E, and F) and metastatic lymph nodes' (G, H, I, J, K, and L) epithelial–mesenchymal transition (EMT) marekrs E-cadherin (A, D, G, and J), N-cadherin (B, E, H, and K), and vimentin (C, F, I, and L) expression.

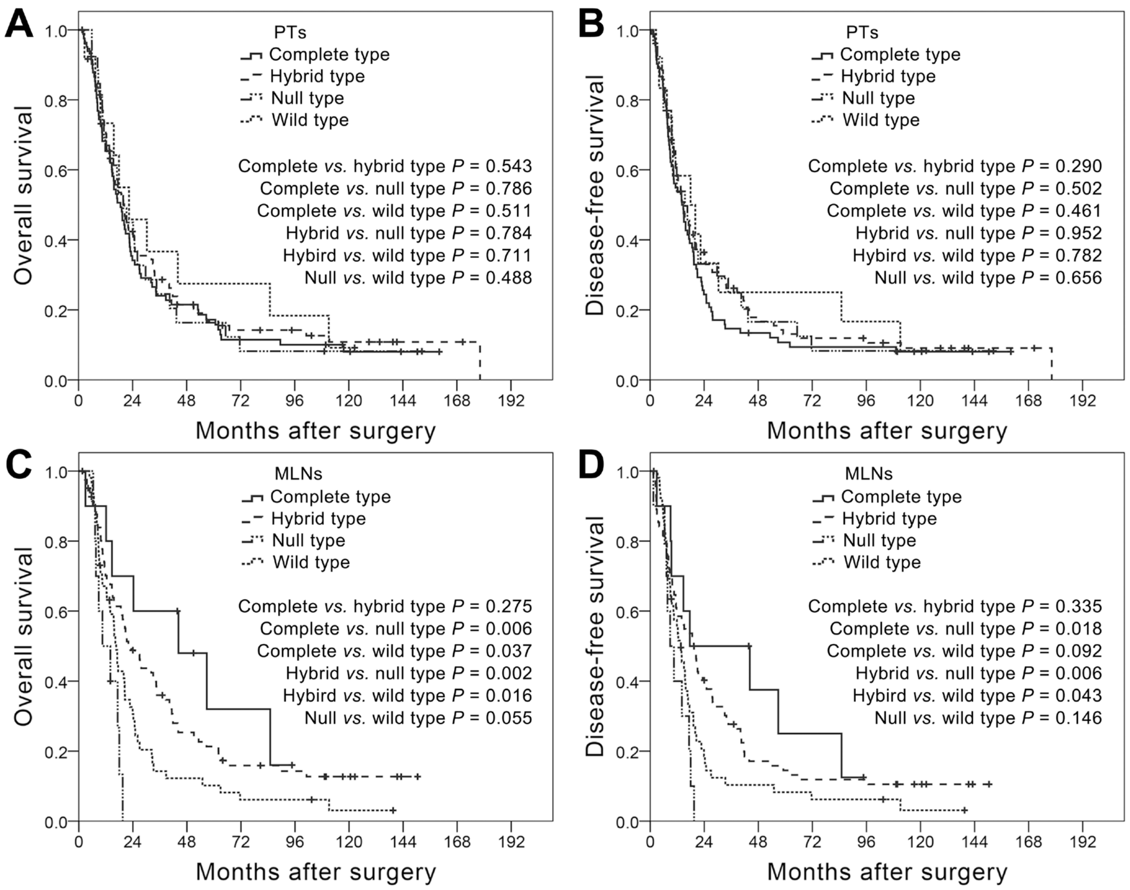

Supplementary Figure S2: Overall survival A. and **C.** and disease-free survival **B.** and **D.** curves by Kaplan–Meier analysis with log-rank test for T3N1-3M0 esophageal squamous cell carcinoma patients according to primary tumors (PTs)' (A and B) or metastatic lymph nodes (MLNs)' (C and D) epithelial–mesenchymal transition (EMT) phenotype classification.

Supplementary Table S1: Differential E-cadherin, N-cadherin and vimentin expression between paired primary tumors (PTs) and metastatic lymph nodes (MLNs) in 155 Stage T3N1-3M0 ESCCs

	Total cases (%)	Cases with different protein expression status at paired PTs and MLNs (PT/MLN)				Cases with discordant protein expression at PTs and MLNs (%)	*P*-value[a]
		+/+ (%)	+/- (%)	-/+ (%)	-/- (%)		
E-Cadherin	155 (100)	59 (38.1)	11 (7.1)	76 (49.0)	9 (5.8)	88 (56.7)	< 0.001
N-Cadherin	155 (100)	47 (30.3)	57 (36.8)	12 (7.7)	39 (25.2)	69 (44.5)	< 0.001
Vimentin	155 (100)	23 (14.8)	44 (28.4)	32 (20.6)	56 (36.1)	76 (49.0)	0.036

[a]Mc-Nemar Chi-square test.

+, positive expression; -, negative expression.

Supplementary Table S2: Relationship between E-cadherin, N-cadherin, and vimentin expression at primary tumors or metastatic lymph nodes and various clinicopathological factors in T3N1-3M0 esophageal squamous cell carcinomas

See supplementary File 1

Supplementary Table S3: Relationship between epithelial-mesenchymal transition phenotypes of primary tumors or metastatic lymph nodes and various clinicopathological factors in T3N1-3M0 esophageal squamous cell carcinomas

See supplementary File 2

Supplementary Table S4: Univariate analysis of primary tumors' and metastatic lymph nodes' epithelial-mesenchymal transition phenotypes and clinicopathological factors for overall survival and disease-free survival in Stage T3N1-3M0 esophageal squamous cell carcinoma patients

Variables	Cases	Overall survival (months)			Disease-free survival (months)		
		Mean	Median	*P*-value[a]	Mean	Median	*P*-value[a]
PT EMT phenotype				0.875			0.686
Wild type	12	43.0	22.4		36.9	18.0	
Null type	26	34.1	19.8		31.7	15.5	
Hybrid type	92	41.1	20.3		35.4	15.0	
Complete type	83	34.9	18.7		28.3	14.7	
LN EMT phenotype				**0.001**			**0.008**
Wild type	53	25.9	16.6		22.1	13.6	
Null type	10	12.8	10.5		11.1	8.8	
Hybrid type	82	42.4	23.3		34.5	19.4	
Complete type	10	48.5	44.2		40.5	17.6	
Gender				0.571			0.693
Male	176	38.9	17.8		33.3	14.1	
Female	37	36.6	24.2		30.7	19.0	
Age (years)				**0.004**			**0.010**
< 58[b]	98	51.7	20.9		44.7	15.5	
≥ 58	115	27.8	18.9		23.1	14.7	
Location				0.636			0.775
Upper	22	41.8	17.1		39.2	15.0	
Middle	120	40.9	20.3		33.0	15.2	
lower	71	33.5	19.2		30.3	13.6	
Length (cm)				0.988			0.853
≤ 5.0[c]	113	36.7	19.5		30.8	15.7	
> 5.0	100	38.4	19.2		33.2	12.5	
Differentiation				0.196			**0.046**
Well	44	52.4	23.0		51.4	20.0	
Moderate	104	36.4	20.2		28.2	13.9	
Poor	65	31.5	16.6		27.2	14.7	
N-stage				**0.009**			**0.010**
N1	122	45.7	21.7		39.1	17.1	
N2-3	91	28.4	157		24.1	13.6	

[a]Kaplan-Meier method, log-rank test.
[b]Median age.
[c]Median length.
PT, primary tumor; MLN, metastatic lymph node; EMT, epithelial-mesenchymal transition.
