# Supplementary material for: The epithelial-mesenchymal transition phenotype of metastatic lymph nodes impacts the prognosis of esophageal squamous cell carcinoma patients
Source: Oncotarget. 2016 Apr 27;7(25):37581–8. doi: 10.18632/oncotarget.9036 (PMC5122333; doi:10.18632/oncotarget.9036)
Supplement: Supplementary file 2 [file oncotarget-07-37581-s002.docx]

**Supplementary Table S2.** Relationship between E-cadherin, N-cadherin, and vimentin expression at primary tumors or metastatic lymph nodes and various clinicopathological factors in T3N1-3M0 esophageal squamous cell carcinomas

|  | Primary tumors | | | | | | | | | | Metastatic lymph nodes | | | | | | | | | |
| --- | --- | --- | --- | --- | --- | --- | --- | --- | --- | --- | --- | --- | --- | --- | --- | --- | --- | --- | --- | --- |
|  | Total | E-Cadherin | | | N-Cadherin | | | Vimentin | | | Total | E-Cadherin | | | N-Cadherin | | | Vimentin | | |
|  |  | - | + | *P* | - | + | *P* | - | + | *P* |  | - | + | *P* | - | + | *P* | - | + | *P* |
| Gender |  |  |  | 0.455*^a^* |  |  | 0.663*^a^* |  |  | 0.758*^a^* |  |  |  | 0.858*^b^* |  |  | 0.258*^a^* |  |  | 0.393*^a^* |
| Male | 176 | 88 | 88 |  | 60 | 116 |  | 100 | 76 |  | 130 | 16 | 114 |  | 78 | 52 |  | 82 | 48 |  |
| Female | 37 | 21 | 16 |  | 14 | 23 |  | 20 | 17 |  | 25 | 4 | 21 |  | 18 | 7 |  | 18 | 7 |  |
| Age (years) |  |  |  | 0.157*^a^* |  |  | 0.989*^a^* |  |  | 0.953*^a^* |  |  |  | 0.426*^a^* |  |  | 0.868*^a^* |  |  | 0.548*^a^* |
| < 58^b^ | 98 | 45 | 53 |  | 34 | 64 |  | 55 | 43 |  | 67 | 7 | 60 |  | 41 | 26 |  | 45 | 22 |  |
| ≥ 58 | 115 | 64 | 51 |  | 40 | 75 |  | 65 | 50 |  | 88 | 13 | 75 |  | 55 | 33 |  | 55 | 33 |  |
| Location |  |  |  | 0.720*^a^* |  |  | 0.954*^a^* |  |  | 0.952*^a^* |  |  |  | 0.729*^a^* |  |  | 0.964*^a^* |  |  | 0.384*^a^* |
| Upper | 22 | 13 | 9 |  | 7 | 15 |  | 12 | 10 |  | 14 | 2 | 12 |  | 9 | 5 |  | 10 | 4 |  |
| Middle | 120 | 61 | 59 |  | 42 | 78 |  | 67 | 53 |  | 90 | 10 | 80 |  | 55 | 35 |  | 54 | 36 |  |
| lower | 71 | 35 | 36 |  | 25 | 46 |  | 41 | 30 |  | 51 | 8 | 43 |  | 32 | 19 |  | 36 | 15 |  |
| Length (cm) |  |  |  | 0.550*^a^* |  |  | 0.347*^a^* |  |  | 0.925*^a^* |  |  |  | 0.078*^a^* |  |  | 0.253*^a^* |  |  | 0.641*^a^* |
| ≤ 5.0*^c^* | 113 | 60 | 53 |  | 36 | 77 |  | 64 | 49 |  | 80 | 14 | 66 |  | 53 | 27 |  | 53 | 27 |  |
| > 5.0 | 100 | 49 | 51 |  | 38 | 62 |  | 56 | 44 |  | 75 | 6 | 69 |  | 43 | 32 |  | 47 | 28 |  |
| Differentiation |  |  |  | 0.402*^a^* |  |  | 0.943*^a^* |  |  | 0.162*^a^* |  |  |  | 0.195*^a^* |  |  | 0.661*^a^* |  |  | 0.193*^a^* |
| Well | 44 | 26 | 18 |  | 16 | 28 |  | 24 | 20 |  | 34 | 4 | 30 |  | 19 | 15 |  | 18 | 16 |  |
| Moderate | 104 | 49 | 55 |  | 35 | 69 |  | 65 | 39 |  | 77 | 7 | 70 |  | 48 | 29 |  | 50 | 27 |  |
| Poor | 65 | 34 | 31 |  | 23 | 42 |  | 31 | 34 |  | 44 | 9 | 35 |  | 29 | 15 |  | 32 | 12 |  |
| N-stage |  |  |  | 0.075*^a^* |  |  | 0.858*^a^* |  |  | 0.141*^a^* |  |  |  | 0.745*^a^* |  |  | 0.881*^a^* |  |  | 0.897*^a^* |
| N1 | 122 | 56 | 66 |  | 43 | 79 |  | 74 | 48 |  | 75 | 9 | 66 |  | 46 | 29 |  | 48 | 27 |  |
| N2-3 | 91 | 53 | 38 |  | 31 | 60 |  | 46 | 45 |  | 80 | 11 | 69 |  | 50 | 30 |  | 52 | 28 |  |

*^a^*Chi-square test.

*^b^*Chi-square test with continuity correction.

+, positive expression; -, negative expression.
