# Supplementary material for: The epithelial-mesenchymal transition phenotype of metastatic lymph nodes impacts the prognosis of esophageal squamous cell carcinoma patients
Source: Oncotarget. 2016 Apr 27;7(25):37581–8. doi: 10.18632/oncotarget.9036 (PMC5122333; doi:10.18632/oncotarget.9036)
Supplement: Supplementary file 3 [file oncotarget-07-37581-s003.docx]

**Supplementary Table S3.** Relationship between epithelial-mesenchymal transition phenotypes of primary tumors or metastatic lymph nodes and various clinicopathological factors in T3N1-3M0 esophageal squamous cell carcinomas

| Variables | Primary tumors | | | | | | Metastatic lymph nodes | | | | | |
| --- | --- | --- | --- | --- | --- | --- | --- | --- | --- | --- | --- | --- |
|  | Total  n (%) | Wild,  n (%) | Null,  n (%) | Hybrid, n (%) | Complete, n (%) | *P*-value | Total  n (%) | Wild,  n (%) | Null,  n (%) | Hybrid,  n (%) | Complete, n (%) | *P*- value |
| Gender |  |  |  |  |  | 0.894*^a^* |  |  |  |  |  | 0.442*^a^* |
| Male | 176 | 10 (5.7) | 21  (11.9) | 78  (44.3) | 67  (38.1) |  | 130 | 42  (32.3) | 8  (6.2) | 72  (55.4) | 8  (6.2) |  |
| Female | 37 | 2  (5.4) | 5  (13.5) | 14  (37.8) | 16  (43.2) |  | 25 | 11  (44.0) | 2  (8.0) | 10  (40.0) | 2  (8.0) |  |
| Age (years) |  |  |  |  |  | 0.318*^b^* |  |  |  |  |  | 0.786*^a^* |
| < 58*^c^* | 98 | 5  (5.1) | 13  (13.3) | 48  (49.0) | 32  (32.7) |  | 67 | 22  (32.8) | 4  (6.0) | 38  (56.7) | 3  (4.5) |  |
| ≥ 58 | 115 | 7  (6.1) | 13  (11.3) | 44  (38.3) | 51  (44.3) |  | 88 | 31  (35.2) | 6  (6.8) | 44  (50.0) | 7  (8.0) |  |
| Location |  |  |  |  |  | 0.811*^a^* |  |  |  |  |  | 0.813*^a^* |
| Upper | 22 | 0  (0) | 4  (18.2) | 9  (40.9) | 9  (40.9) |  | 14 | 5  (35.7) | 1  (7.1) | 7  (50.0) | 1  (7.1) |  |
| Middle | 120 | 6  (5.0) | 15  (12.5) | 53  (44.2) | 46  (38.3) |  | 90 | 29  (32.2) | 4  (4.4) | 51  (56.7) | 6  (6.7) |  |
| Lower | 71 | 6  (8.5) | 7  (9.9) | 30  (42.3) | 28  (39.4) |  | 51 | 19  (37.3) | 5  (9.8) | 24  (47.1) | 3  (5.9) |  |
| Length (cm) |  |  |  |  |  | 0.695*^c^* |  |  |  |  |  | 0.223*^a^* |
| ≤ 5.0*^d^* | 113 | 6  (5.2) | 12  (10.6) | 47  (41.6) | 48  (42.5) |  | 80 | 28 (35.0) | 8  (10.0) | 6  (7.5) | 8  (10.0) |  |
| > 5.0 | 100 | 6  (6.0) | 14  (14.0) | 45  (45.0) | 35  (35.0) |  | 75 | 25  (33.3) | 2  (2.7) | 44  (58.7) | 4  (5.3) |  |
| Differentiation |  |  |  |  |  | 0.619*^b^* |  |  |  |  |  | 0.278*^a^* |
| Well | 44 | 2  (4.5) | 4  (9.1) | 16  (36.4) | 22  (50.0) |  | 34 | 10  (29.4) | 1  (2.9) | 20  (58.8) | 3  (8.8) |  |
| Moderate | 104 | 6 (5.8) | 15  (14.4) | 49 (47.1) | 34  (32.7) |  | 77 | 25  (32.5) | 4  (5.2) | 45  (58.4) | 3  (3.9) |  |
| Poor | 65 | 4  (6.2) | 7  (10.8) | 27  (41.5) | 27  (41.5) |  | 44 | 18  (40.9) | 5  (11.4) | 17  (38.6) | 4  (9.1) |  |
| N-stage |  |  |  |  |  | **0.039***^b^* |  |  |  |  |  | 0.951*^a^* |
| N1 | 122 | 9  (7.4) | 18  (14.8) | 57  (46.7) | 38  (31.1) |  | 75 | 25  (33.3) | 4  (5.3) | 41  (54.7) | 5  (6.7) |  |
| N2-3 | 91 | 3  (3.3) | 8  (8.8) | 35  (38.5) | 45  (49.5) |  | 80 | 28  (35.0) | 6  (7.5) | 41  (51.3) | 5  (6.3) |  |
| Relapse pattern after surgery |  |  |  |  |  | 0.899*^a^* |  |  |  |  |  | 1.000*^a^* |
| Local recurrence | 42 | 2  (4.8) | 5  (11.9) | 19  (45.2) | 16  (38.1) |  | 32 | 11  (34.4) | 1  (3.1) | 18  (56.3) | 2  (6.3) |  |
| Distant organ metastasis | 19 | 1  (5.3) | 1  (5.3) | 8  (42.1) | 9  (47.4) |  | 15 | 5  (33.3) | 0  (0) | 9  (60.0) | 1  (6.7) |  |

*^a^*Fishers’ exact test.

*^b^*Chi-square test.

*^c^*Median age.

*^d^*Median length.
